# Atrial Thrombus or Atrial Myxoma? Preliminary Analysis of Echocardiographic Findings of a Case Series

**DOI:** 10.2174/011573403X281926240417110330

**Published:** 2024-04-25

**Authors:** Guobing Hu, Fang Song

**Affiliations:** 1 Department of Ultrasound, the First Affiliated Hospital of Wannan Medical College, Wuhu 241001, Anhui, China

**Keywords:** Thrombus, echocardiography, myxoma, atrial fibrillation, heart diseases

## Abstract

**Background:**

Echocardiography has been proven to be a useful tool for detecting atrial-occupying lesions, ranging from primary or secondary tumors to thrombi. Although the precise diagnosis is important as clinical treatment modalities differ, sometimes differentiating a thrombus from a myxoma is very difficult.

**Case Report:**

From January 2019 to December 2022, we retrospectively analyzed the echocardiographic findings of 8 patients who were found to have an interatrial mass. Of the 8 patients, 4 had a right atrial mass, and 4 had a left atrial mass. Based on ultrasonic examination, the initial diagnosis was a thrombus and the second diagnosis was a myxoma for all 8 patients. All masses were finally confirmed to be thrombi. Although an echocardiogram can provide significant information on the nature of atrial masses in many patients, qualitative diagnosis of a small percentage of atrial masses remains difficult.

**Conclusion:**

An atrial thrombus is occasionally difficult to differentiate from an atrial myxoma in patients without atrial fibrillation, especially when it is not attached to the left atrial appendage. Upon review of the echocardiographic findings of the 8 patients described in our study, it is essential to highlight the fact that a thrombus can mimic a myxoma and thereby create a diagnostic conundrum.

## 1 INTRODUCTION

An atrial thrombus is frequently associated with atrial fibrillation, while an atrial myxoma is not always associated with atrial fibrillation. Although previous studies have demonstrated that echocardiography may be a sensitive and effective tool for detecting atrial-occupying lesions, sometimes neither Transthoracic Echocardiography (TTE) nor Transesophageal Echocardiography (TEE) leads to a definitive diagnosis. Very little literature has been devoted to the reasons for misdiagnosis of echocardiographic findings. Here, we have mainly discussed the echocardiographic features of interatrial masses of 8 patients in the hope of avoiding misdiagnosis in the future.

## 2 PATIENTS AND METHODS

From January 2019 to December 2022, 8 patients were diagnosed by echocardiography as having an interatrial mass; a first diagnosis of a thrombus and a second diagnosis of a myxoma were both made in the ultrasonic examination report. Atrial fibrillation was excluded in all patients, and other associated structural heart diseases were also excluded, as well as all other thrombotic conditions, such as coagulative cascade anomalies, paraneoplastic conditions, *etc*. Of the 8 patients, 4 were male and 4 were female, and their ages were between 50 and 89 years (mean 68.7 years). TTE and TEE were carried out using a Philips IE33 echocardiography system, and a transducer (frequency, 3/3.75/5 MHz) was selected. Our study was approved by the Medical Ethics Committee of Yijishan Hospital. Informed consent was waived by the medical ethics committee due to its retrospective nature. We have de-identified all patient details for the study. The authors certified that all appropriate patient consent forms have been signed. In the form, the patients have given their consent for their images and other clinical information to be reported in the journal. The patients understood that their names and initials would not be published, because efforts would be made to conceal their identity, but anonymity cannot be guaranteed.

In the study period, therapeutic anticoagulation was carried out in all patients. A second echocardiographic examination showed that the atrial mass disappeared in 5 patients; the remaining 3 patients had undergone surgery, and the interatrial mass was pathologically confirmed as a thrombus. There were no other associated cardiac diseases, such as serious valvular heart diseases, pulmonary heart diseases, or other congenital heart diseases. The echocardiographic findings, clinical history, and clinical treatment results for the 8 patients are displayed in Table **1**, and other TTE findings are shown in Table **2**.

## 3 DISCUSSION

Echocardiography has been proven to be a useful tool for detecting atrial-occupying lesions, ranging from primary or secondary tumors to thrombi. Atrial myxomas have been proven to be the most common benign tumors, with the majority of them originating in the interatrial septum and extending to the left or right atrial chamber, and the remainder originating in the left or right ventricle, superior or inferior vena cava or pulmonary veins. Both right and left atrial myxomas mainly begin in the fossa ovalis [1]. On an echocardiogram, a myxoma is normally pedunculated, a polypoid, oval or round in shape, and lobulated [2, 3]. On gross inspection, myxomas most frequently appear gelatinous.

A mass originating from the left atrial appendage is easily diagnosed as a thrombus in patients with atrial fibrillation [4, 5]. Reportedly, thrombi are not enhanced unless they are vascularized in rare cases; in contrast, myxomas are always inhomogeneously enhanced [6]. The presence of vascularity alone should not be used to differentiate a thrombus from a myxoma because neovascularization is not pathognomonic for intracardiac tumors [7]. Regardless of whether the intracardiac mass is a myxoma or thrombus, there is a major risk of embolism or even sudden death if the mass ruptures.

Sometimes differentiating a thrombus from a myxoma is very difficult, and precise diagnosis is important as clinical treatment modalities differ. A myxoma should be surgically removed, whereas a thrombus might be absolutely dissolved with anticoagulation [8]. Reportedly, organized thrombi and myxomas cannot be differentiated on cardiac magnetic resonance images due to the heterogeneity of signal intensity and the presence of vascularization [9-11]. Some cardiologists suggest that Pulsed Wave (PW) Tissue Doppler Imaging (TDI) might be useful in differentiating pathological cardiac masses from pseudo-masses. Indeed, the pathological ones have a dyssynchronous motion, not concordant with surrounding structures, whereas the pseudo masses have a concordant motion with surrounding myocardial tissue [12].

**Fig. (1):**
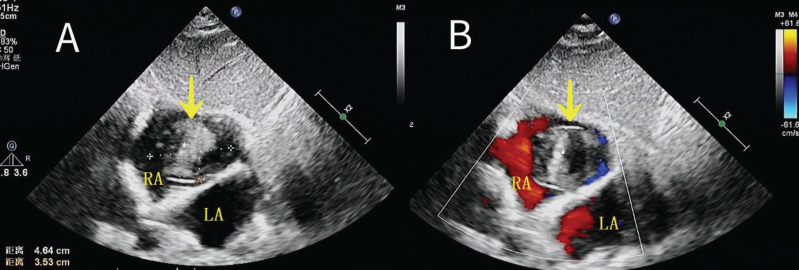
(**A, B**) A 46×35 mm mobile mass in the right atrium attached to the Interatrial Septum (IAS).

**Fig. (2):**
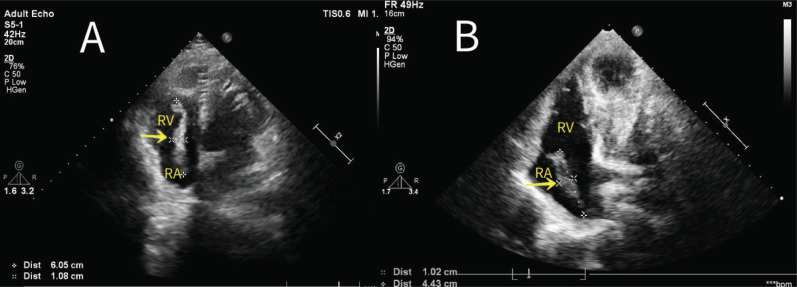
(**A**, **B**) A mobile squirrel tail-like mass arising from the IAS near the inferior vena cava.

In our study, atrial fibrillation was excluded in all patients; 4 patients had a mass originating from the Interatrial Septum (IAS), 2 patients had a mobile squirrel tail-like mass arising from the interatrial septum near the inferior vena cava, and 2 patients had a mass attached to the lateral wall of the left atrium (Figs. **1** and **2**). Upon review of the echocardiographic findings of the 8 patients described in our study, it is essential to highlight the fact that a thrombus can mimic a myxoma and thereby create a diagnostic conundrum. In the case of atypical echocardiographic features of an atrial mass, a thrombus should be considered. If a mass can be dissolved with anticoagulants, then it is likely a thrombus; otherwise, the mass should be removed surgically and confirmed by histopathologic examination. We summarize the difficulties in qualitative diagnosis as follows: (1) atrial fibrillation was excluded in all patients; (2) none of the masses in our study originated from the left or right atrial appendage; and (3) all masses were flexible and mobile.

## CONCLUSION

An atrial thrombus is occasionally difficult to differentiate from an atrial myxoma in patients without atrial fibrillation, especially when it is not attached to the left atrial appendage. The limitation of our study is that we did not have an adequate size sample of patients. Thus, the echocardiographic findings of 8 patients are not enough to provide novel guidance on the differential diagnosis of atrial masses. Therefore, a larger sample of patients is needed.

## Figures and Tables

**Table 1:** Echocardiographic findings and clinical results.

**Case** **No.**	**Age** **(Year)**	**Sex**		**Clinical History**	**Echocardiographic Findings**	**Clinical Treatment Results**
1	70	F	The patient presented with progressive exertional breathlessness and palpitations for 5 days. She was hemodynamically stable. Previously, she had been in good health except for mildly elevated blood pressure.		A 46×35 mm mobile hyperechoic mass in the right atrium was pedunculated and attached to interatrial septum (IAS) (Fig. **1**), in view of its relation to IAS, thepossibility of myxoma was retanied.	Operative findings showed the mass adherent to the inte-atrial septum; on gross inspection, it appeared to be a thrombus; histopathologic examination confirmed an old organizing thrombus in various stages of organization comprised of fibrin and degenerating inflammatory cells, with no evidence of myxoma.
2	57	M	The patient was admitted to our hospital with a 2-month history of progressive precordial discomfort and palpitation.		A 42×31 mm oval heterogenous mass with a short stalk bulging out from IAS in the right atrium, The mass was flexible and mobile.	Operative findings showed the mass to be densely adherent to IAS in the right atrium. Frozen section analysis confirmed a thrombus. Subsequent histopathological examination of multiple sections of the mass confirmed an organized thrombus with vascularization confined mostly to the periphery; no myxoma cell were seen
3	50	M	The patient had undergone open surgery for left kidney calculi two years prior, he was then in a good state with no complications. A mass was detected during a routine checkup by TTE.		A mobile squirrel tail-like mass arising from IAS near the inferior vena cave; the mass was 61 mm in length, and 9-11 mm in width (Fig. **2**).	Operative findings showed the mass to be adherent to the IVS near the inferior vena cava, histopathologic examination confirmed an organized thrombus, with no evidence of myxoma,.
4	68	M	The patient presented with progressive exertional dyspnea for two weeks. He had undergone mitral valvoplasty four years prior.		A 28*25 mm oval-shaped hyperechoic mass with central necrosis attached to IAS in the left atrium; the mass was flexible, mobile and pedunculated.	The patient was started on oral anticoagulation, heparinization was administered, and no mass was found one week later on TTE.
5	77	F	The patient was admitted to our hospital with a 1-week history of dizziness and unstable walking. Left middle cerebral artery occlusion was diagnosed and a large amount of thrombus tissue was extracted by catheter.		A 33*26 mm irregular hyperechoic mass attached by a narrow base to the lateral wall of the left atrium, the mass was flexible and mobile.	Heparinization was administered, and no mass was found ten days later on TTE.
6	89	F	The patient was admitted to our hospital with a 1-month history of chest tightness		A 38×28 mm mobile irregular left atrial mass with a relative broad base arising from IAS near the root of anterior mitral leaflet.	Anticoagulation was carried out; on the seventh day of admission, a second echocardiographic examination showed no mass in the left atrium
7	66	M	The patient had a low fever for three months.		A mobile squirrel tail-like mass arising from IAS near the inferior vena cava. The mass was 62 mm in length; and 6-11 mm in width.	After one-week treatment with anticoagulation, the mass had disappeared and could not be seen on TTE.
8	73	F	The patient was admitted to our hospital with sudden dysphasia and left limb weakness for 4 hours.		A 40×20 mm mobile irregular left atrial mass with a relative broad base arising from the lateral wall of the left atrium.	After one-week thrombolytic therapy and anti-coagulation, the mass had disappeared.

**Abbreviation:** IAS, inter-atrial septum.

**Table 2:** Other TTE findings.

**Case No.**	**Age (Year)**	**Sex**	**LA (mm)**	**LVDD (mm)**	**RA (mm)**	**RV (mm)**	**EF**	**Other Abnormal Findings**
1	70	F	32	42	53*41	21	59%	Mild aortic valve regurgitation
2	57	M	37	46	57*40	23	60%	Mild tricuspid valve regurgitation
3	50	M	38	49	47*36	22	66%	-
-4	68	M	32	49	43*34	19	31%	-
5	77	F	44	50	56*40	20	58%	Mild mitral valve regurgitation
6	89	F	48	42	55*42	23	59%	Moderate mitral valve regurgitation
7	54	M	35	43	47*36	22	66%	-
8	73	F	31	47	44*34	21	66%	-

**Abbreviation:** LA, left atrium; LVDD, left ventricle diastolic diameter ; RA, right atrium; RV right ventricle; EF, ejection fraction.

## Data Availability

The authors confirm that the data supporting the findings of this research are available within the article.

## LIST OF ABBREVIATIONS

TTE: Transthoracic Echocardiography
TEE: Transesophageal Echocardiography

## STANDARDS OF REPORTING

STROBE guidelines were followed.
